# Multimodal deep learning of fundus abnormalities and traditional risk factors for cardiovascular risk prediction

**DOI:** 10.1038/s41746-023-00748-4

**Published:** 2023-02-02

**Authors:** Yeong Chan Lee, Jiho Cha, Injeong Shim, Woong-Yang Park, Se Woong Kang, Dong Hui Lim, Hong-Hee Won

**Affiliations:** 1 Department of Digital Health, Samsung Advanced Institute for Health Sciences & Technology (SAIHST), Sungkyunkwan University, Samsung Medical Center, Seoul, Republic of Korea; 2 Research Institute for Future Medicine, Samsung Medical Center, Seoul, Republic of Korea; 3 Graduate School of Future Strategy, Korea Advanced Institute of Science and Technology (KAIST), Daejeon, Republic of Korea; 4 Samsung Genome Institute, Samsung Medical Center, Sungkyunkwan University School of Medicine, Seoul, Republic of Korea; 5 Department of Ophthalmology, Samsung Medical Center, Sungkyunkwan University School of Medicine, Seoul, Republic of Korea

**Keywords:** Risk factors, Cardiovascular diseases, Cerebrovascular disorders, Predictive markers

## Abstract

Cardiovascular disease (CVD), the leading cause of death globally, is associated with complicated underlying risk factors. We develop an artificial intelligence model to identify CVD using multimodal data, including clinical risk factors and fundus photographs from the Samsung Medical Center (SMC) for development and internal validation and from the UK Biobank for external validation. The multimodal model achieves an area under the receiver operating characteristic curve (AUROC) of 0.781 (95% confidence interval [CI] 0.766–0.798) in the SMC and 0.872 (95% CI 0.857–0.886) in the UK Biobank. We further observe a significant association between the incidence of CVD and the predicted risk from at-risk patients in the UK Biobank (hazard ratio [HR] 6.28, 95% CI 4.72–8.34). We visualize the importance of individual features in photography and traditional risk factors. The results highlight that non-invasive fundus photography can be a possible predictive marker for CVD.

## Introduction

Cardiovascular disease (CVD) accounts for an estimated 31% of deaths worldwide, and 17.3 million people die from CVDs each year. Despite global efforts in public health and increasing medical knowledge, the high burden of CVD rarely decreases because of complicated risk factors that require long-term behavioral and pharmaceutical modifications^1^. Although no single cost-effective screening and detection tool is clinically adaptable to predict CVDs, risk assessment tools such as the Framingham risk score and the European systematic coronary risk evaluation are well-established, with statistical modeling, that combines traditional risk factors such as age, sex, total and high-density lipoprotein (HDL) cholesterol, blood pressure, smoking, and diabetes^2–4^. Persistent efforts have been made in recent decades to improve risk prediction models by identifying and reclassifying risk factors^5,6^.

Recent systematic reviews have shown that traditional cardiovascular risk assessment can over- or under-predict CVD risks, and it has limited benefits on patient outcomes^7^. Even now, identifying the best risk assessment models is difficult given the differences in risk categories, the availability of comparable cohorts, and heterogeneity in at-risk populations^8,9^. Promising biomarkers directly related to inflammation and atherosclerotic burdens, such as the ankle-brachial index, high-sensitivity C-reactive protein, and coronary artery calcium (CAC) score, have been suggested recently; however, these nontraditional models have rarely shown a significant improvement in CVD predictions^8^.

The non-invasive visualization of atherosclerotic vascular abnormalities, such as cardiac computed tomography or carotid ultrasound imaging, is one of the most accurate clinical assessments for patients with low to intermediate pre-test CVD probability^10–13^. However, routine screening of the coronary or carotid arteries has been discouraged because of its low clinical- and cost-effectiveness in patients with lower risk factors^14^. In contrast, fundus photographs (FP) are widely used in health screening examinations for ocular diseases because they are cost-effective. Furthermore, FP provide more information to assess CVD risk, including non-invasive visualization of atherosclerotic vascular abnormalities. The pathologic relationship between retinal microvascular changes and systemic vascular abnormalities has been well-recognized^15–18^.

Although a clinical guideline for diagnosing CVD through FP has not yet been established, artificial intelligence approaches have shown FP to predict CVD-related biomarkers as well as CVD events^19–23^. Rim et al. suggested that FP predicted a CAC score, and Poplin et al. showed that FP could be a predictor of cardiovascular risk factors, including age, sex, smoking status, and systolic blood pressure, and of major adverse cardiac events. They also sought to predict future cardiovascular events. Diagnosing risk factors for CVD using non-invasive and cost-effective predictive models is also important. This is because, in low- and middle-income countries where laboratory facilities and clinicians are limited and the CVD burden is increasing^24^, such predictive models are particularly promising for an accurate diagnosis of CVD and the identification of high-risk individuals.

This study aimed to develop and evaluate an automated method to predict current CVDs, as defined by International Classification of Diseases (ICD) codes, using FP. We simultaneously compared the results with traditional risk scores for CVD prediction. Using multimodal deep learning, this study attempted to combine retinal fundus abnormalities from FP with traditional epidemiological risk factors for better CVD prediction. To our knowledge, both traditional ophthalmoscopy and recently digitalized retinal photography have not been clinically validated as standardized techniques to identify CVD^15,25^. Clinical studies investigating the relationship between retinal microvascular abnormalities and CVDs are mostly limited to retinal image data with clinical labels or FP with specific grading of vascular atherosclerotic abnormalities^15,18^. Meanwhile, automated assessment of retinal images using a deep convolutional neural network analysis does not require manual feature extraction, such as grading, thus enabling the analysis of large image data without losing diverse patterns of retinal abnormalities, including vasculature. Furthermore, the multimodal deep learning method has potential benefits in simultaneously quantifying CVD risks from FP along with the clinical risk factors used in traditional risk assessment tools.

## Results

We used 1758 images for CVD cases and 1760 images for non-CVD controls from the Samsung Medical Center (SMC) for model development. For model validation, we used 1421 images for CVD cases and 1533 images for non-CVD controls from SMC, and 613 images for CVD cases and 10,685 images for controls from the UK Biobank. The basic patient characteristics are summarized in Table 1. The non-CVD controls were randomly undersampled, corresponding to the number of cases (1758 and 1421 images) in the development and internal validation sets, respectively. The proportion of patients with diabetes and hypertension in the SMC was much higher than in the UK Biobank. Our model achieved a receiver operating characteristic curve (AUROC) of up to 0.905 (95% confidence interval [CI], 0.891–0.919) using multimodal data for external validation.

**Table 1:** Baseline characteristics of the participants.

	Development set (SMC)	Internal validation set (SMC)	*p*-value	External validation set (UK Biobank)
**No. of images**			0.141	
CVD case	1758 (50.0%)	1421 (48.1%)		613 (5.4%)
Non-CVD control	1760 (50.0%)	1533 (51.9%)		10,685 (94.6%)
**No. of patients**	2,026	517		11,091
Sex			0.922	
Male	1085 (53.6%)	275 (53.2%)		5044 (45.5%)
Female	941 (46.4%)	242 (46.8%)		6047 (54.5%)
Age (years)	61.5 ± 12.6	62.1 ± 12.6	0.402	57.3 ± 8.2
Systolic blood pressure	124.3 ± 17.4	125.0 ± 17.9	0.424	139.4 ± 19.3
Total cholesterol	170.5 ± 37.8	171.1 ± 39.8	0.772	221.1 ± 43.8*
HDL cholesterol	54.1 ± 15.5	53.9 ± 15.7	0.776	58.0 ± 15.1*
Diabetes	951 (46.9%)	265 (51.3%)	0.088	603 (5.4%)
Hypertension	952 (47.0%)	249 (48.2%)	0.083	876 (7.9%)

*The unit (mmol/dl) of cholesterol in the external validation set was converted to mg/dl. (1 mg/dl = 1 mmol/dl × 38.67).

*HDL* High-density lipoprotein, *SMC* Samsung Medical Center.

### Improvements through the addition of FPs

**Fig. 1:**
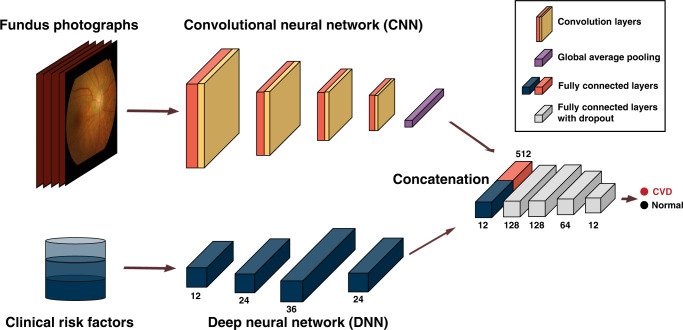
Illustration of multimodal architecture which combines CNN with DNN to feed fundus photographs and clinical risk factors. DenseNet-169 architecture with convolution layers (i.e., dense blocks) is used for CNN. The number of nodes is specified at each fully-connected layer. The last three layers have a dropout rate of 0.3. DNN deep neural network, CNN convolutional neural network.

**Fig. 2:**
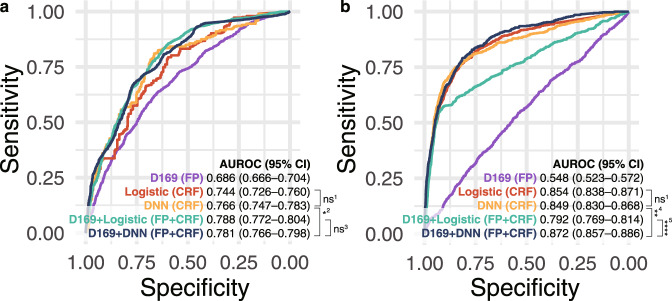
Prediction performance of the models. Receiver operating characteristic curves in the internal validation set (**a**) and external validation set (**b**). The performances of the models (Models 1–5) are described in Table 2. ^1^The differences in AUROCs between the logistic regression and the DNN are not significant (*p*-value = 0.079 in **a** and *p*-value = 0.531 in **b**). ^2^The difference in AUROCs between the DNN and the multimodal network is marginally significant (*p*-value = 0.047). ^3^The difference in AUROCs between the ensemble model (D169 + logistic regression) and the multimodal network is not significant (*p*-value = 0.541). ^4^The difference in AUROCs between the DNN and the multimodal network is significant (*p*-value = 0.004). ^5^The difference in AUROCs between the ensemble model (D169 + logistic regression) and the multimodal network is significant (*p*-value < 2 × 10^−16^). AUROC area under the receiver operating characteristic curve, CI confidence interval, D169 DenseNet-169, FP fundus photographs, Logistic logistic regression, CRF clinical risk factors, CRF, DNN deep neural network.

We present the indices of the models in Table 2 for brevity (Models 1–11). Our multimodal networks (Models 5, 8–11) were developed using FP and clinical risk factors (CRF), whereas deep neural networks (DNN) (Models 3 and 7) were developed using only CRF (Fig. 1). The receiver operating characteristic (ROC) curves of the models are shown in Fig. 2. The difference in the AUROCs between logistic regression and the DNN was not statistically significant. However, the AUROC of 0.781 (95% CI 0.766–0.798) for the multimodal network (Model 5) was significant and marginally greater than that of 0.766 (95% CI 0.747–0.783) for the DNN (Model 3) in internal validation (Table 2 and Fig. 2a; p-value = 0.047). The multimodal model (Model 5) achieved an accuracy, sensitivity, specificity, positive predictive value (PPV), and negative predictive value (NPV) of 0.683, 0.871, 0.508, 0.621, and 0.810, respectively, whereas the DNN (Model 3) achieved accuracies, sensitivities, specificities, PPV, and NPV of 0.719, 0.749, 0.691, 0.692, and 0.748, respectively (Supplementary Tables 1-2). Additionally, we evaluated the DNN according to latent periods of 0 and 3 months to define CVD cases (Supplementary Table 3), and we observed similar performances with the main model using a latent period of 6 months. An ensemble model (Model 4) of CNN for FP and logistic regression for CRF achieved an AUROC of 0.788 (95% CI 0.772–0.804), which was not significantly different from that of the multimodal network (Model 5) (Fig. 2a; p-value = 0.541).

**Table 2:** Area under the receiver operating characteristic curves in the internal validation set and the external validation set.

Index	Model	Variables	AUROC (95% CI)	
			Internal validation set (SMC)	External validation set (UK Biobank)
1	DenseNet-169	FP	0.686 (0.666–0.704)	0.548 (0.523–0.572)
2	Logistic regression	CRF	0.744 (0.726–0.760)	0.854 (0.838–0.871)
3	DNN	CRF	0.766 (0.747–0.783)	0.849 (0.830–0.868)
4	DenseNet-169 + Logistic regression	FP + CRF	0.788 (0.772–0.804)	0.792 (0.769–0.814)
5	DenseNet-169 + DNN	FP + CRF	0.781 (0.766–0.798)	0.872 (0.857–0.886)
6	10-year ASCVD risk of PCE^4^	CRF + smoking*	—	0.677 (0.658–0.696)
7	DNN	CRF	^a^	0.854 (0.839–0.867)
8	DenseNet-169 + DNN	FP + CRF	^a^	0.882 (0.868–0.895)
9	DenseNet-169 + DNN	FP + non-invasive CRF	^a^	0.830 (0.812–0.847)
10	DenseNet-169 + DNN + Uncertainty^b^	FP + CRF	^a^	0.905 (0.891–0.919)
11	DenseNet-169 + DNN + Uncertainty^b^	FP + non-invasive CRF	^a^	0.898 (0.877–0.915)

Clinical risk factors (CRF): Sex + Age + SBP + TC + HDL + Diabetes + Hypertension.

Non-invasive clinical risk factors: Sex + Age + SBP + Diabetes + Hypertension.

*We additionally add smoking history to calculate the risk; however, we cannot conduct the risks in SMC because the variable was not available.

^a^We used all multimodal data in SMC (development set + internal validation set) as training data for improving the model performance. Therefore, AUROCs in SMC were not measured.

^b^We considered the predictions with high confidence according to 95% uncertainty intervals.

*AUROC* area under the receiver operating characteristics curve, *SMC* Samsung Medical Center, *FP* fundus photographs, *ASCVD* atherosclerotic cardiovascular disease, *PCE* Pooled Cohort Equation, *DNN* deep neural network, *FP* fundus photographs, SBP systolic blood pressure, *T*C total cholesterol, *HDL* high-density lipoprotein cholesterol.

**Fig. 3:**
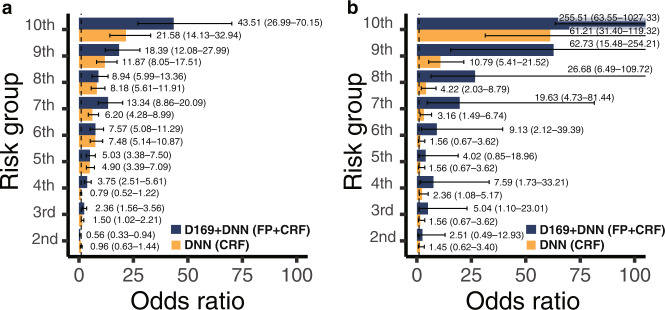
Risk gradient for CVD according to the risk group. Odds ratios with 95% confidence interval (error bar) according to the risk groups in comparison to the lowest risk group in the internal validation set (**a**) and external validation set (**b**). D169 + DNN and DNN are identical in Model 5 and Model 3 in Table 2, respectively. CVD cardiovascular disease, D169 DenseNet-169, DNN deep neural network, FP fundus photographs, CRF clinical risk factors.

The predicted risk scores of Models (Model 3 and 5) were associated with the prevalence of CVD (Fig. 3a). The odds ratios (ORs) between the highest and lowest risk groups derived from the predicted probability were 43.51 (95% CI 26.99–70.15) and 21.58 (95% CI 14.13–32.94) for the multimodal network and DNN, respectively. We observed an increasing pattern of ORs in all risk groups in both models. All ORs of the multimodal network were higher than the ORs of the DNN in each risk group, except for the second group in the DNN.

We externally tested the performance of an independent set of UK Biobanks. In total, 11,091 participants from the UK Biobank were selected for testing. Our multimodal model (Model 5) achieved an AUROC of 0.872 (95% CI 0.857–0.886), which was significantly greater than the AUROC of 0.849 (95% CI 0.830–0.868) for the DNN (Model 3) (Table 2 and Fig. 2b; *p*-value = 0.004). The AUROC of the multimodal network (Model 5) was significantly higher than the AUROC of the ensemble model (Model 4) of CNN and logistic regression (Fig. 2b; *p*-value < 2 × 10^−16^). The ORs in the UK Biobank showed a pattern similar to that of the ORs in the SMC (Fig. 3b). Most ORs for the multimodal network (Model 5) were higher than those for DNN (Model 3). When the multimodal model was trained with all SMC data, the AUROC in the external validation increased further (Model 8 vs. Model 5).

### Comparisons between CRF and non-invasive CRF

**Fig. 4:**
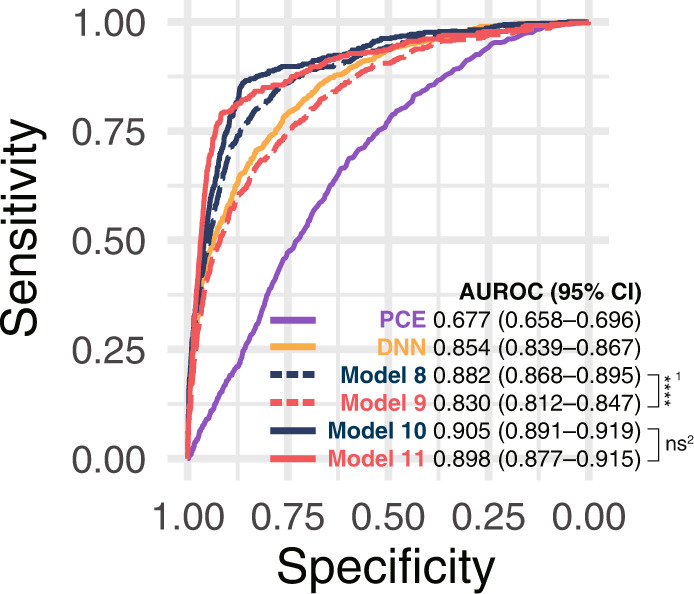
Receiver operating characteristic curves in the external validation set. The performances of the models (Models 6–11) are described in Table 2. The models were trained with multimodal data in the SMC dataset. ^1^The difference in AUROCs between Models 8 and 9 is significant (p-value < 2 × 10^−16^). ^2^The difference in AUROCs between Models 10 and 11 is not significant (*p*-value = 0.578). AUROC Area under the receiver operating characteristics curve, PCE Pooled Cohort Equation, DNN deep neural network, CRF clinical risk factors, SMC Samsung Medical Center.

We compared multimodal networks, including CRF (Model 8) and non-invasive CRF (Model 9), trained with all SMC data. Non-invasive CRF included sex, age, systolic blood pressure (SBP), diabetes, and hypertension. Additionally, we calculated the 10-year atherosclerotic cardiovascular disease (ASCVD) risk of Pooled Cohort Equation (PCE)^4^ (Model 6) in the external validation set (AUROC 0.677, 95% CI, 0.658–0.696). Figure 4 and Table 2 show the AUROC of the models. The multimodal network trained with FP and CRF (Model 8) was significantly higher than the network trained with FP and non-invasive CRF (Model 9) (*p*-value < 2 × 10^−16^). In addition, for patients classified as having or not having CVD with confidence using uncertainty quantification, Models 10 and 11 achieved AUROCs of nearly 0.9, with no significant difference (*p*-value = 0.578). Model 10 predicted 591 CVD cases out of 9,116 participants with confidence, whereas Model 11 predicted 420 CVD cases out of 7219 participants.

### Association between the predicted scores and future CVD events in at-risk patients

**Fig. 5:**
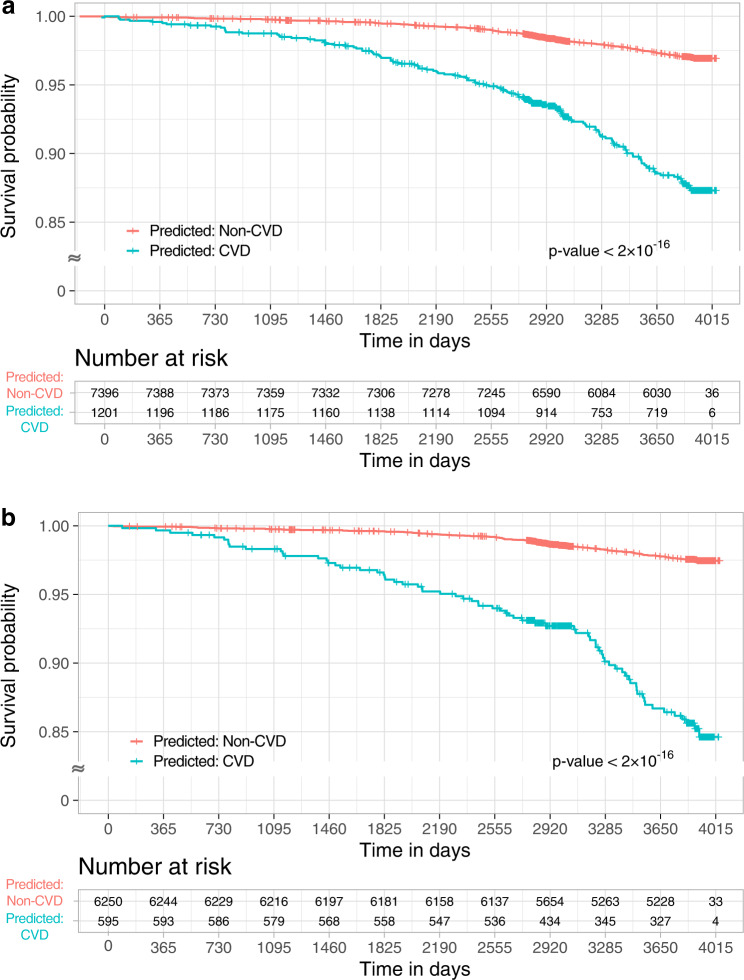
Association of predicted risk with incident CVD. Kaplan–Meier graphs for incident CVD in the at-risk patients according to the predicted class from Model 10 (**a**) and Model 11 (**b**). CVD cardiovascular disease.

We investigated the incidence of CVD in at-risk patients based on predicted scores. We selected 10,784 multimodal data points from 10,588 at-risk patients. The predicted scores of Models 8 and 9 were statistically associated with the incidence of CVD in at-risk patients (Supplementary Table 4 and Supplementary Figs. 2–3). We additionally stratified at-risk patients by 10-year ASCVD risk (Supplementary Table 5). At-risk patients categorized as CVD cases and those with a high 10-year ASCVD risk had an increased risk of CVD (Table 3; hazard ratio [HR] = 5.24 [95% CI 4.02–6.82] for Model 9). In addition, the effect of the multimodal network on CVD was prominent in the low-borderline 10-year ASCVD risk group (HR = 2.10 [95% CI 1.24–3.54] for Model 9). In Models 10 and 11, with uncertainty quantification, the risk of CVD was also significantly increased in at-risk patients who were predicted to have CVD (Fig. 5 and Supplementary Tables 6–7; HR = 6.28 [95% CI 4.72–8.34] for Model 11).

**Table 3:** Hazard ratios (95% CI) for future CVD events of subgroups by the multimodal network (Models 8–9) and 10-year ASCVD risk with at-risk patients in the UK Biobank.

		10-year ASCVD risk		
Variables (Model)	Risk group	Low-borderline	Intermediate	High
FP + CRF (Model 8)	Predicted: Non-CVD	1 (Reference)	2.50 (1.93–3.23)	2.59 (1.64–4.09)
	Predicted: CVD	3.37 (1.93–5.88)	3.85 (2.92–5.07)	6.08 (4.63–8.00)
FP + non-invasive CRF (Model 9)	Predicted: Non-CVD	1 (Reference)	2.11 (1.53–2.89)	1.90 (0.84–4.33)
	Predicted: CVD	2.10 (1.24–3.54)	3.39 (2.65–4.33)	5.24 (4.02–6.82)

Model 8 was trained with FP and CRF using all multimodal data (development set + internal validation set) in SMC.

Model 9 was trained with FP and non-invasive CRF using all multimodal data (development set + internal validation set) in SMC. Clinical risk factors (CRF): Sex + Age + SBP + TC + HDL + Diabetes + Hypertension.

Non-invasive clinical risk factors: Sex + Age + SBP + Diabetes + Hypertension.

*CI* Confidence interval, *CVD* cardiovascular disease, *ASCVD* Atherosclerotic cardiovascular disease, *FP* fundus photographs.

### Model interpretation

**Fig. 6:**
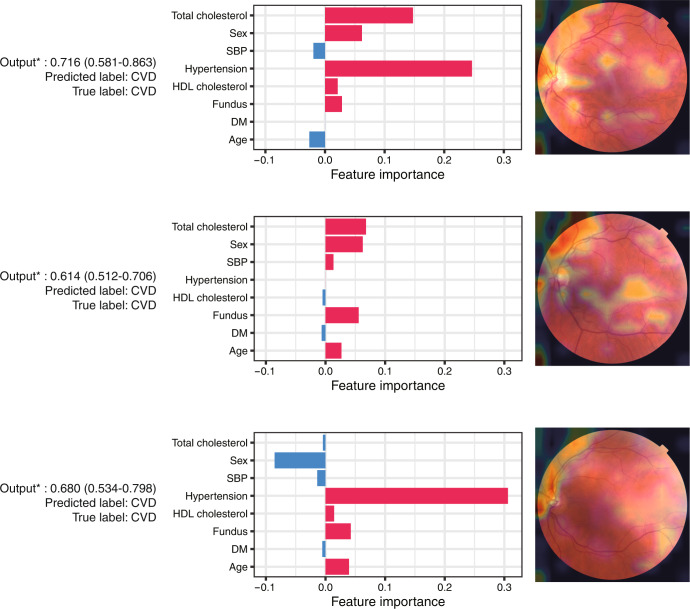
Examples of true positives in the external validation for interpretation. The predicted scores (output) are described with the probability of the sample and 95% uncertainty interval (left). The SHAP values for contributions of variables are depicted in the middle. Positive feature importance (red) indicates that the variable may be a risk factor for predicting CVD, whereas a negative (blue) variable indicates a protective feature. Heatmap generation with fundus photographs using Grad-CAM (right). CVD cardiovascular disease, Grad-CAM gradient-weighted class activation mapping, SHAP Shapley additive explanation.

Our multimodal network can be interpreted using uncertainty quantification^26^, Shapley additive explanation (SHAP)^27^, and gradient-weighted class activation mapping (Grad-CAM)^28^. Fig. 6 shows three randomly selected true positives with interpretations in the external validation set. In the first example, the model predicted an output of 0.716 (95% uncertainty interval; 0.581–0.863). Because the interval did not include a classification threshold of 0.5, the prediction was considered to be confident. The input variables were then interpreted with SHAP values indicating the feature importance. Hypertension showed the greatest feature importance in predicting CVD. Finally, Grad-CAM, which is based on the attention mechanism, highlighted the optic disc and blood vessels. The important features that contribute to CVD prediction vary among patients.

## Discussion

We developed a multimodal deep learning model for CVD risk prediction using two independent datasets. We designed an integrated deep-learning network that combines two networks using two different modalities: FP and CRF. A convolutional neural network (CNN) with state-of-the-art deep learning architecture was constructed for imaging data, and a simple DNN was developed for CRF. Using internal and external validation sets, we confirmed the predictive performance of the multimodal network and DNN. Retinal fundus photography is a good, non-invasive indicator of microvascular changes. However, a recent study developed an image-only model that achieved an AUROC of 0.70, which warrants further studies to improve the prediction performance^20^. The retinal fundus has the potential to assess biomarkers, such as C-reactive protein^19^.

CRFs, such as age, sex, SBP, total cholesterol, HDL cholesterol, diabetes, and hypertension, are well-known cardiovascular risk factors. An AUROC of 0.73 to predict major adverse cardiac events in a multimodal model developed by Poplin et al. indicated that using various factors can lead to better predictive results^20^. Our results also showed that FP can be a cardiovascular risk factor in diagnosing CVD at present, and a multimodal model can obtain significantly higher performance than a model with a single modality (Table 2 and Figs. 2, 3). This pattern was more pronounced in our multimodal network than in the DNN, when externally validated using the UK Biobank. Interestingly, the AUROCs in the UK Biobank were much higher than those of SMC. Patients with SMC who visited the Department of Ophthalmology could have more underlying diseases than those in the UK Biobank, who were considered generally healthier participants. Racial differences may also affect these results. We developed the models using a balanced dataset and externally validated them using a highly imbalanced dataset. This variance may have affected the differences in performance. Moreover, we presented the potential of a multimodal network trained only with FP and non-invasive CRF. The AUROC of Model 11 with non-invasive CRF and FP using uncertainty was not significantly different from the AUROC of Model 10 using invasive clinical measurements. Prediction with non-invasive CRF may be essential for diagnosing CVD in resource-limited clinical environments. Furthermore, our results highlighted a significant association between the incidence of future CVD events and predicted scores. The results indicate that our multimodal models also have the potential to predict future CVD events.

Previous studies^18,29–32^ have shown that retinal microvascular abnormalities are directly associated with an increased CVD risk; however, they have not been clinically evaluated. Nonetheless, deep learning to extract more detailed information from fundus images to predict CVD risk determination has also been applied^19,20,33–35^. Interestingly, Rim et al. developed a deep learning model to predict cardiovascular risk using CAC scores from retinal photographs^19^. They showed the potential of retinal photographs as a predictor of CVD. In other studies, cardiovascular risk factors, including age and sex, were associated with retinal FP^20,21^. Traditionally, other studies have been conducted to predict CVD using conventional risk factors^36–38^. other biomarkers have been discovered to diagnose CVD^39^. In contrast, current state-of-the-art algorithms enable us to develop a model with multimodal data. We focused on predicting CVD at present to help diagnose patients with easily accessible medical information. We also suggest that the predicted scores from our model were associated with future CVD events. Our multimodal model, trained with FP, CRF, and non-invasive CRF, demonstrated improvement in the reclassification of cases and controls when a new modality (images) was combined (Table 3). Our results suggest that this approach may help predict and prevent complex diseases such as CVD.

Our study had several limitations. First, we defined CVD cases as participants diagnosed with CVD at a single medical center. This operational definition could be biased because some patients may have been diagnosed with CVDs in other medical centers. However, we externally validated our models using the UK Biobank. Second, we trained our model using data from a relatively small sample. We excluded participants with missing retinal fundus images or electronic medical records (EMR). However, for generalizability, we pretrained 30,000 unlabeled images with unsupervised learning and assigned a sufficiently large portion to the test data. The number of samples in the training set was almost identical to that in the internal validation set. Furthermore, we observed consistent results using the UK Biobank as an external validation set. Third, the dataset used for model development did not represent the true incidence of CVD. We set the threshold for classification to 0.5 in the development. We applied an identical threshold in the external validation, which was imbalanced in terms of the CVD outcome. The model predicted an unseen patient, regardless of the distribution. Furthermore, we observed consistent results in the imbalanced dataset (613 CVD cases vs. 10,685 non-CVD controls). Fourth, the participants whose data were used for training our model originated from a single country, but the external validation performed in another country was consistent. In future studies, training a model with an integrated dataset or transfer learning from a large dataset to the target dataset may improve prediction performance. Fifth, this study had a retrospective design. Our model showed that trained features from a retinal fundus image are associated with cardiovascular events and could be a new cardiovascular risk factor. Nonetheless, we observed an association between the predicted score and future CVD events. Using the concept of multimodal deep learning, future work is required to develop and evaluate advanced multimodal artificial intelligence models with various types of data such as imaging, EMR, biological signals, and environmental factors. It is also important to find the optimal model using deep learning in addition to recent automated machine learning methods^40,41^. Furthermore, adopting such models in the public health system or clinics requires a cost-effectiveness analysis for comparisons of CRF and non-invasive CRF models.

In conclusion, our findings suggest that the retinal fundus can be used as a biomarker to predict cardiovascular risk using two independent datasets. We showed improved results, including the retinal fundus, for classifying the CVD cases. Future studies are needed to develop a multimodal model using non-invasive factors and to determine the clinical implications in retinal FPs.

## Methods

This study used a multimodal deep learning method to predict CVD and simultaneously assessed its performance with clinical CVD risk factors in the validation sets.

Ethical approval for the study protocol was granted by the Institutional Review Board (No:2016-05-561) of the SMC. The use of The UK Biobank was approved by the National Research Ethics Committee (REC Reference 11/NW/0382). Data from the SMC were collected retrospectively and anonymized at the clinical data warehouse of the SMC. We used data from the SMC of patients who provided informed consent for the research. All the participants in the UK Biobank provided written informed consent.

### Subjects

Patients who visited the SMC between March 2010 and May 2016 with available FP were included in the study. Retinal FP and matched EMR were obtained retrospectively. Patients with retinal or vitreous pathology that affects the detection of microvascular changes in the FP (e.g., retinal detachment, macular degeneration, or vitreous hemorrhage) were excluded. The fundus images were reviewed by an ophthalmologist, and those of retinal diseases or of low quality, including blurred, artifactual, underexposed, and very low-contrast resolution images, were removed from the development set. Additionally, diagnostic codes (*International Classification of Diseases*, 10th Revision, ICD-10, H33 and H353, H431) were applied to exclude FP of retinal diseases. The flow diagrams of the study population are shown in Supplementary Fig. 1.

According to the diagnosis code in the EMR, CVD cases were defined as adults (≥18 years old) who visited our clinic with coronary heart disease (ICD-10, I20–I25) or cerebrovascular disease (ICD-10, I63–I69) at least 6 months before the retinal FP was taken. Non-CVD controls were defined as adults without CVD. We set a latent period of 6 months because it was assumed to be the time period between symptom presentation and diagnosis.

### Datasets

We selected 2543 patients with both FP and EMR from 37,395 patients with retinal FP in the SMC between 2010 and 2016. Patients in the non-CVD control group were randomly selected for under-sampling to prevent overfitting. Participants were randomly divided into two groups for model development and validation: 3518 FP and EMR obtained from 2026 patients for development and 2954 FP and EMR obtained from 517 patients for internal validation. Additional independent retinal images with incomplete EMR were used to pre-train the deep learning model. EMR that were within 6 months from the date of the fundus examination were used to collect data on seven clinical CVD risk factors used in the conventional CVD risk tool^4^. Data on five of the risk factors including sex, age, systolic blood pressure, diabetes, and hypertension were collected non-invasively from the EMR. The remaining risk factors, total and HDL cholesterol, were measured in the blood samples. The risk factors were selected on the basis of a guideline for the assessment of the CVD risk developed by the American College of Cardiology (ACC) and the American Heart Association (AHA)^4^. Among the clinical predictors, we normalized age, systolic blood pressure, total, and HDL cholesterol levels. Retinal image data and seven structured clinical datasets were simultaneously used to train the deep multimodal network. Patients with missing data were excluded.

To test for external validity, we selected eligible, de-identified participants from approximately 500,000 participants in a prospective cohort, the UK Biobank (Supplementary Fig. 1). For external validation, UK Biobank participants who had FP and EMR were defined in the same way as described above. We further included patients diagnosed with coronary heart disease and cerebrovascular disease with ICD-9 codes 410–412 and 433–438, respectively. Finally, 11,298 FP and EMR were obtained from 11,091 patients.

### Image preprocessing

All retinal images were captured using a fundus camera (TRC-50DX Retinal Camera; Topcon Corporation, Tokyo, Japan). The full-resolution images were converted to JPEG with center cropping, random flipping horizontally and vertically, random rotation within 30° for data augmentation, random brightness with an adjustment factor of 0.1, and random zoom with an adjustment factor of 0.1; they were then converted to a resolution of 448 × 448 pixels for use in the neural network. All images and EMR from eligible patients were identified before their transfer to data analysts.

### Model development

In this study, we used a multimodal deep learning method, which is an algorithm that trains networks with multiple sources. A multimodal network was created to combine a CNN for retinal images and a DNN for clinical risk factors (Fig. 1).

We used the DenseNet-169 architecture to analyze the image data, which has good performance with fewer parameters compared with other CNN architectures^42^. DenseNet-169 comprises five convolution layers and four dense blocks including 164 convolution layers. In addition, we attached it to fully connected layers for concatenation. To improve classification performance, we pretrained the convolutional neural network using unsupervised learning. We constructed an autoencoder with a U-Net architecture, including the DenseNet architecture as an encoder and convolution layers with a skip connection as a decoder. The autoencoder was trained to predict an FP as close as possible to itself. We used the pretrained DenseNet architecture as part of the multimodal network.

We used a DNN to learn the nonlinear interactions among CRF. The network had five fully connected layers followed by a rectified linear unit. The weights of the networks were randomly initialized.

These two separate networks were then concatenated into four fully connected layers with a dropout rate of 0.3. The final layer with a sigmoid function was added to perform the disease classification. The binary cross-entropy loss of the model was minimized with stochastic gradient descent with a learning rate of 0.001 dropped 0.9 times at every 10 epochs and trained in 20 epochs with a batch size of 4.

### Model evaluation

We assessed the area under the AUROC to classify patients with and without disease, based on predictive values. We obtained 95% confidence intervals for the AUROC with 1000 bootstrap samples. DeLong’s tests were performed to statistically compare the AUROCs. Additionally, the sensitivity, specificity, PPV and NPV were used for evaluation. To compare the prediction performance between the risk assessment models with and without retinal images, multivariable logistic regression was performed with the prediction scores from the multimodal network. We stratified the prediction scores into deciles and estimated the ORs of each decile in comparison with the lowest decile.

We calculated the 10-year ASCVD risk using the PCE for comparison with our models^4^. The 10-year risk for ASCVD was categorized according to the definition given by the American College of Cardiology Foundation. We obtained an AUROC of the 10-year ASCVD risk in the UK Biobank.

In the UK Biobank, we further confirmed that the predicted score is associated with the incidence of CVD in at-risk patients. At-risk patients were defined as those who were not diagnosed with CVD at least 6 months before the FP. We conducted log-rank tests for the incidence of CVD by categorizing the risk groups according to the predicted scores for at-risk patients and plotted the survival probability using Kaplan-Meier curves. Cox proportional hazards analysis was performed to estimate HRs and 95% CIs. The categories were low (bottom 20%), high (top 20%), and intermediate (others), according to the predicted score. From another perspective, at-risk patients can also be divided into two groups: patients who were predicted to have CVD and those who were predicted to be non-CVD controls.

We used TensorFlow (version 2.9.0, Google, Mountain View, CA, USA) to train the deep learning models and R software (version 3.6.3, Vienna, Austria) for data processing and evaluation. Statistical significance was set at *p* < 0.05.

### Model interpretation

We used three approaches to interpret our model; uncertainty quantification^26^, SHAP^27^, and Grad-CAM^28^. First, uncertainty quantification indicates how confident the model is for a given prediction. Using our model, we estimated the uncertainty interval of each test sample with 100 repetitions. The interval was calculated with an equal-tailed credible interval with a confidence level of 0.05; *I*_0.05_ = [*q*_0.03_, *q*_0.98_] where *q*_*n*_ is the *n*th percentile of the posterior distribution. We assumed that the classification of the output was confident if the 95% uncertainty interval for the output did not include a classification threshold of 0.5. Second, we used a framework for interpreting predictions using SHAP to provide a level of feature importance for each prediction. The SHAP values are the Shapley values of conditional expectation, which demonstrate the contributions of each individual variable. Finally, we used Grad-CAM to visualize the highlighted important regions for the model to predict the target^28^. The Grad-CAM uses gradient information from the last convolutional layer with back-propagation. The gradients were averaged globally to calculate the importance of the feature maps to a target. The estimated importance of the feature map represents the confidence score of the model.

### Reporting summary

Further information on research design is available in the Nature Research Reporting Summary linked to this article.

## Supplementary information

Supplementary Information

Reporting Summary

## Data Availability

Not all SMC data are publicly available because of restrictions on data sharing. The UK Biobank data are available with a proper application process at https://www.ukbiobank.ac.uk/enable-your-research/apply-for-access. An interested researcher can contact the corresponding author for additional information.

## Code availability

The DenseNet code used in this study is available at https://github.com/liuzhuang13/DenseNet. The source code and pretrained models for this study are available to the corresponding author upon academically reasonable request.
