# Supplementary material for: Multimodal deep learning of fundus abnormalities and traditional risk factors for cardiovascular risk prediction
Source: NPJ Digit Med. 2023 Feb 2;6:14. doi: 10.1038/s41746-023-00748-4 (PMC9894867; doi:10.1038/s41746-023-00748-4)

Supplementary Information

Multimodal deep learning of fundus abnormalities and traditional risk factors for cardiovascular risk prediction

Yeong Chan Lee[*], Jiho Cha[*], Injeong Shim, Woong-Yang Park, Se Woong Kang, Dong Hui Lim[#], Hong-Hee Won[#]

[*]These authors contributed equally to this work as co-first authors.

[#]These authors contributed to this work as co-corresponding authors.

Co-corresponding author: **Hong-Hee Won, Ph.D.**

Samsung Advanced Institute for Health Sciences and Technology (SAIHST), Sungkyunkwan University, Samsung Medical Center, 81 Irwon-ro, Gangnam-gu, Seoul 06351, Republic of Korea

Phone: +82-2-2148-7566; Fax: +82-2-3410-0534; E-mail: wonhh@skku.edu

Co-corresponding author: **Dong Hui Lim, M.D., Ph.D.**

Department of Ophthalmology, Samsung Medical Center, Sungkyunkwan University School of Medicine, Seoul, Republic of Korea

Phone: +82-2-3410-3568; Fax: +82-2-3410-0074; E-mail: donghui.lim@samsung.com

Contents

Supplementary Table 1. Performances of models in the internal validation set 3

Supplementary Table 2. Performances of models in the external validation set................................ 4

Supplementary Table 3. Area under the receiver operating characteristic curves of the DNN trained with FP 5

Supplementary Table 4. Hazard ratios (95% CI) for CVD by the multimodal networks in the UK Biobank 6

Supplementary Table 5. Hazard ratios (95% CI) for CAD of 10-year ASCVD risk for at-risk patients in the UK Biobank 7

Supplementary Table 6. Hazard ratios (95% CI) for CVD by the multimodal networks (Models 10-11) in the UK Biobank 8

Supplementary Table 7. Hazard ratios (95% CI) for future CVD events of subgroups by the multimodal network (Models 10-11) and 10-year ASCVD risk in the UK Biobank 9

Supplementary Figure 1. Flow diagrams of the study populations of the (a) Samsung Medical Center and (b) UK Biobank. 10

Supplementary Figure 2. Kaplan–Meier graphs for incident CVD in the external validation according to the predicted score (a) and the predicted class (b) of Model 8.. 11

Supplementary Figure 3. Kaplan–Meier graphs for incident CVD in the external validation according to the predicted score (a) and the predicted class (b) of Model 9.. 12

Supplementary Table 1. Performances of models in the internal validation set.

Index	Model	Variables	Accuracy	Sensitivity	Specificity	PPV	NPV
1	DenseNet-169	FP	0.639	0.592	0.682	0.633	0.643
2	Logistic regression	CRF	0.680	0.669	0.690	0.667	0.692
3	DNN	CRF	0.719	0.749	0.691	0.692	0.748
4	DenseNet-169 + Logistic regression	FP + CRF	0.715	0.716	0.714	0.699	0.731
5	DenseNet-169 + DNN	FP + CRF	0.683	0.871	0.508	0.621	0.810

Clinical risk factors (CRF): Sex + Age + SBP + TC + HDL + Diabetes + Hypertension.
Non-invasive clinical risk factors: Sex + Age + SBP + Diabetes + Hypertension.
We used all multimodal data in SMC (development set + internal validation set) as training data for improving the model performance. Therefore, AUROCs in SMC were not measured.
We considered the predictions with high confidence according to 95% uncertainty intervals.
Abbreviations: positive predictive value, PPV; negative predictive value, NPV; deep neural network, DNN; fundus photographs, FP; systolic blood pressure, SBP; total cholesterol, TC; high-density lipoprotein cholesterol, HDL.

Supplementary Table 2. Performances of models in the external validation set.

Index	Model	Variables	Accuracy	Sensitivity	Specificity	PPV	NPV
1	DenseNet-169	FP	0.067	0.979	0.015	0.054	0.923
2	Logistic regression	CRF	0.921	0.485	0.946	0.342	0.970
3	DNN	CRF	0.881	0.692	0.891	0.268	0.981
4	DenseNet-169 + Logistic regression	FP + CRF	0.186	0.951	0.142	0.060	0.981
5	DenseNet-169 + DNN	FP + CRF	0.471	0.941	0.444	0.088	0.992
7	DNN[#]	CRF	0.733	0.802	0.729	0.145	0.985
8	DenseNet-169 + DNN[#]	FP + CRF	0.788	0.835	0.786	0.183	0.988
9	DenseNet-169 + DNN[#]	FP + non-invasive CRF	0.663	0.830	0.653	0.121	0.985
10	DenseNet-169 + DNN[#] + Uncertainty[†]	FP + CRF	0.863	0.859	0.863	0.269	0.991
11	DenseNet-169 + DNN[#] + Uncertainty[†]	FP + non-invasive CRF	0.909	0.788	0.915	0.33	0.988

Clinical risk factors (CRF): Sex + Age + SBP + TC + HDL + Diabetes + Hypertension.
Non-invasive clinical risk factors: Sex + Age + SBP + Diabetes + Hypertension.
10-year ASCVD risk of PCE[4] provided four risk categories with a risk score; therefore, these binary metrics were not conducted.
[#]We used all multimodal data in SMC to improve the models.
[†]We considered the predictions with high confidence according to 95% uncertainty intervals.
Abbreviations: positive predictive value, PPV; negative predictive value, NPV; deep neural network, DNN; fundus photographs, FP; systolic blood pressure, SBP; total cholesterol, TC; high-density lipoprotein cholesterol, HDL.

Supplementary Table 3. Area under the receiver operating characteristic curves of the DNN trained with FP.

Latent period	Model	Variables	AUROC (95% CI) Internal validation set (SMC)	AUROC (95% CI) External validation set (UK Biobank)
0 month	DenseNet-169	FP	0.662 (0.643–0.682)	0.535 (0.511–0.559)
3 months	DenseNet-169	FP	0.635 (0.616–0.655)	0.555 (0.531–0.578)
6 months	DenseNet-169	FP	0.686 (0.666–0.704)	0.548 (0.523–0.572)

Abbreviations: deep neural network, DNN; fundus photographs, FP; area under the receiver operating characteristics curve, AUROC; Samsung Medical Center, SMC.

Supplementary Table 4. Hazard ratios (95% CI) for CVD by the multimodal networks in the UK Biobank.

Model	Risk group	Patients	Incidence rate (1,000 PYs)	Hazard ratio (95% CI)
Model 8	Low (<20%)	2,159	1.71	1 (Reference)
	Intermediate (20-80%)	6,472	3.57	2.15 (1.52–3.03)
	High (>80%)	2,155	9.39	5.90 (4.16–8.35)
	Predicted: Non-CVD	8,288	3.02	1 (Reference)
	Predicted: CVD	2,498	8.73	3.02 (2.51–3.63)
Model 9	Low (<20%)	2,156	1.56	1 (Reference)
	Intermediate (20-80%)	6,476	3.62	2.40 (1.69–3.43)
	High (>80%)	2,154	9.41	6.55 (4.57–9.40)
	Predicted: Non-CVD	6,671	2.52	1 (Reference)
	Predicted: CVD	4,115	7.31	3.05 (2.53–3.69)

Model 8 was trained with FP and CRF using all multimodal data (development set + internal validation set) in SMC.

Model 9 was trained with FP and non-invasive CRF using all multimodal data (development set + internal validation set) in SMC.

Clinical risk factors (CRF): Sex + Age + SBP + TC + HDL + Diabetes + Hypertension.

Non-invasive clinical risk factors: Sex + Age + SBP + Diabetes + Hypertension.

Abbreviations: confidence interval, CI; cardiovascular disease, CVD; person-years, PYs; systolic blood pressure, SBP; total cholesterol, TC; high-density lipoprotein cholesterol, HDL.

Supplementary Table 5. Hazard ratios (95% CI) for CAD of 10-year ASCVD risk for at-risk patients in the UK Biobank.

Risk group	Patients	Incidence rate	Hazard ratio (95% CI)
Low-borderline	5,512	2.17	1 (Reference)
Intermediate	3,868	5.72	2.71 (2.17–3.38)
High	1,406	9.13	4.49 (3.49–5.77)

Supplementary Table 6. Hazard ratios (95% CI) for CVD by the multimodal networks (Models 10-11) in the UK Biobank.

Model	Risk group	Patients	Incidence rate (1,000 PYs)	Hazard ratio (95% CI)
Model 10	Predicted: Non-CVD	7,397	2.74	1 (Reference)
	Predicted: CVD	1,202	11.27	4.39 (3.51–5.48)
Model 11	Predicted: Non-CVD	6,250	2.28	1 (Reference)
	Predicted: CVD	595	13.08	6.28 (4.72–8.34)

Model 10 was trained with FP and CRF using all multimodal data (development set + internal validation set) in SMC and selected the at-risk patients with whom the model was confident.

Model 11 was trained with FP and non-invasive CRF using all multimodal data (development set + internal validation set) in SMC and selected the at-risk patients with whom the model was confident.

Clinical risk factors (CRF): Sex + Age + SBP + TC + HDL + Diabetes + Hypertension.

Non-invasive clinical risk factors: Sex + Age + SBP + Diabetes + Hypertension.

Abbreviations: confidence interval, CI; cardiovascular disease, CVD; person-years, PYs; systolic blood pressure, SBP; total cholesterol, TC; high-density lipoprotein cholesterol, HDL.

Supplementary Table 7. Hazard ratios (95% CI) for future CVD events of subgroups by the multimodal network (Models 10-11) and 10-year ASCVD risk in the UK Biobank.

		**10-year ASCVD risk**		
**Variables (Model)**	**Risk group**	**Low-borderline**	**Intermediate**	**High**
FP + CRF (Model 10)	Predicted: Non-CVD	1 (Reference)	2.42 (1.83-3.21)	2.50 (1.30-4.78)
	Predicted: CVD	6.84 (3.00-15.57)	5.27 (3.82-7.28)	7.44 (5.42-10.21)
FP + non-invasive CRF (Model 11)	Predicted: Non-CVD	1 (Reference)	2.00 (1.39-2.88)	1.00 (0.25-4.04)
	Predicted: CVD	9.07 (3.34-24.62)	6.69 (4.50-9.95)	7.73 (5.26-11.36)

Model 10 was trained with FP and CRF using all multimodal data (development set + internal validation set) in SMC and selected the at-risk patients with whom the model was confident.

Model 11 was trained with FP and non-invasive CRF using all multimodal data (development set + internal validation set) in SMC and selected the at-risk patients with whom the model was confident.

Clinical risk factors (CRF): Sex + Age + SBP + TC + HDL + Diabetes + Hypertension.

Non-invasive clinical risk factors: Sex + Age + SBP + Diabetes + Hypertension.

Abbreviations: confidence interval, CI; cardiovascular disease, CVD; atherosclerotic cardiovascular disease, ASCVD; fundus photographs, FP; systolic blood pressure, SBP; total cholesterol, TC; high-density lipoprotein cholesterol, HDL.

Supplementary Figure 1. Flow diagrams of the study populations of the (a) Samsung Medical Center and (b) UK Biobank. Abbreviations: fundus photographs, FP; electronical medical records, EMR; cardiovascular disease, CVD.

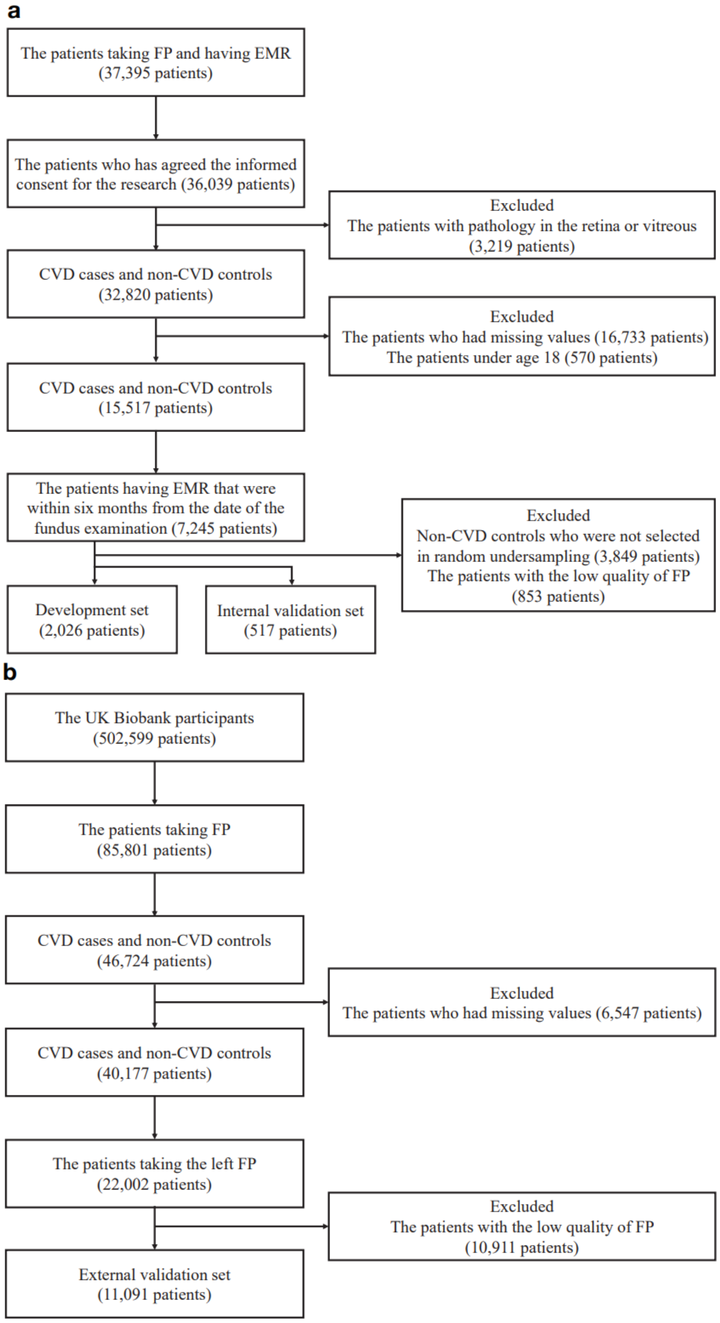

Supplementary Figure 2. Kaplan–Meier graphs for incident CVD in the external validation according to the predicted score (a) and the predicted class (b) of Model 8. Abbreviations: cardiovascular disease, CVD.

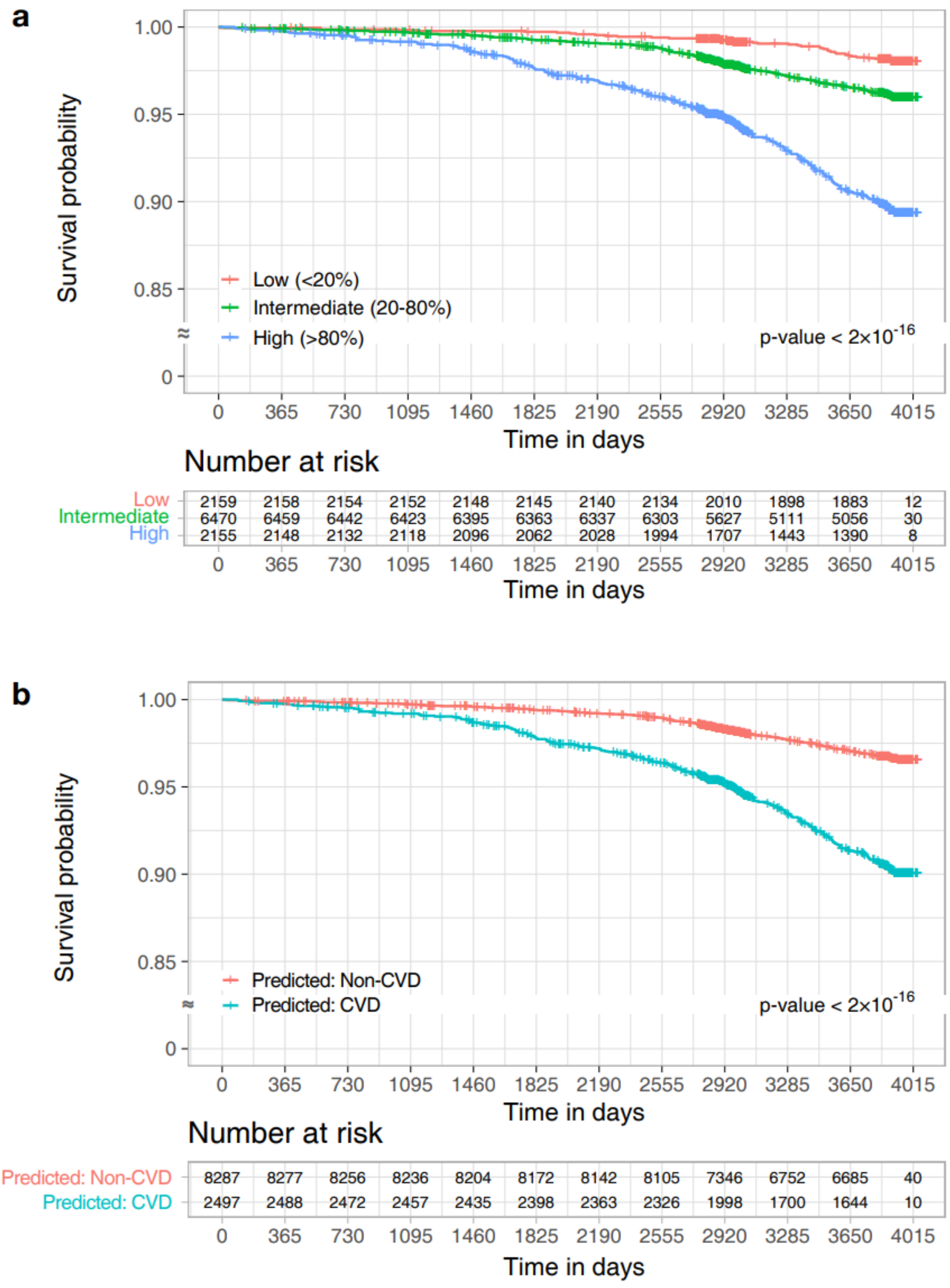

Supplementary Figure 3. Kaplan–Meier graphs for incident CVD in the external validation according to the predicted score (a) and the predicted class (b) of Model 9. Abbreviations: cardiovascular disease, CVD.

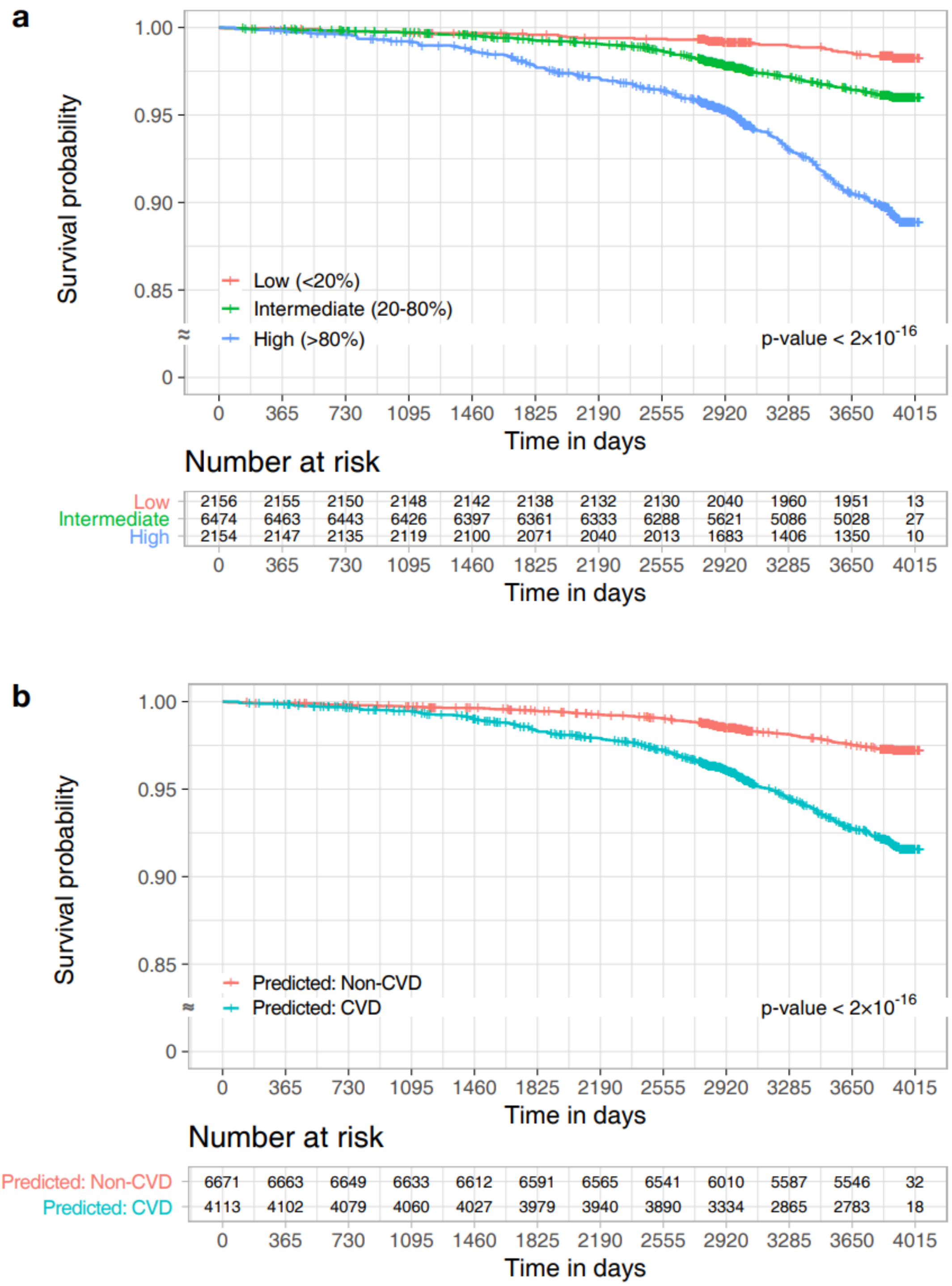

Supplement: Supplementary file 1 — Supplementary Information [file 41746_2023_748_MOESM1_ESM.pdf]
