# Supplementary material for: Multimodal deep learning of fundus abnormalities and traditional risk factors for cardiovascular risk prediction
Source: NPJ Digit Med. 2023 Feb 2;6:14. doi: 10.1038/s41746-023-00748-4 (PMC9894867; doi:10.1038/s41746-023-00748-4)
Supplement: Supplementary file 2 — Reporting Summary [file 41746_2023_748_MOESM2_ESM.pdf]

nature portfolio

Corresponding author(s): Hong-Hee Won

Last updated by author(s): Aug 2, 2022

Reporting Summary

Nature Portfolio wishes to improve the reproducibility of the work that we publish. This form provides structure for consistency and transparency in reporting. For further information on Nature Portfolio policies, see our Editorial Policies and the Editorial Policy Checklist.

Statistics

For all statistical analyses, confirm that the following items are present in the figure legend, table legend, main text, or Methods section.

n/a	Confirmed	
☐	☒	The exact sample size (n) for each experimental group/condition, given as a discrete number and unit of measurement
☐	☒	A statement on whether measurements were taken from distinct samples or whether the same sample was measured repeatedly
☐	☒	The statistical test(s) used AND whether they are one- or two-sided<br>*Only common tests should be described solely by name; describe more complex techniques in the Methods section.*
☐	☒	A description of all covariates tested
☐	☒	A description of any assumptions or corrections, such as tests of normality and adjustment for multiple comparisons
☐	☒	A full description of the statistical parameters including central tendency (e.g. means) or other basic estimates (e.g. regression coefficient) AND variation (e.g. standard deviation) or associated estimates of uncertainty (e.g. confidence intervals)
☐	☒	For null hypothesis testing, the test statistic (e.g. F, t, r) with confidence intervals, effect sizes, degrees of freedom and P value noted<br>*Give P values as exact values whenever suitable.*
☒	☐	For Bayesian analysis, information on the choice of priors and Markov chain Monte Carlo settings
☒	☐	For hierarchical and complex designs, identification of the appropriate level for tests and full reporting of outcomes
☐	☒	Estimates of effect sizes (e.g. Cohen's d, Pearson's r), indicating how they were calculated

Our web collection on statistics for biologists contains articles on many of the points above.

Software and code

Policy information about availability of computer code

Data collection	No software was used to collect the data.
Data analysis	We used TensorFlow (version 2.9.0, Google, Mountain View, CA, USA) to train the deep learning models and used the R software (version 3.6.3, Vienna, Austria) for data processing and result evaluation. Statistical significance was set at p-value < 0.05.

For manuscripts utilizing custom algorithms or software that are central to the research but not yet described in published literature, software must be made available to editors and reviewers. We strongly encourage code deposition in a community repository (e.g. GitHub). See the Nature Portfolio guidelines for submitting code & software for further information.

Data

Policy information about availability of data

All manuscripts must include a data availability statement. This statement should provide the following information, where applicable:

- Accession codes, unique identifiers, or web links for publicly available datasets
- A description of any restrictions on data availability
- For clinical datasets or third party data, please ensure that the statement adheres to our policy

All Samsung Medical Center data are not publicly available because of restrictions for data sharing. The UK Biobank data can be available with a proper application process at https://www.ukbiobank.ac.uk/enable-your-research/apply-for-access.

Human research participants

Policy information about studies involving human research participants and Sex and Gender in Research.

Reporting on sex and gender	We used sex as a biological variable which was self-reported. We did not perform sex-based analyses. We used data from the Samsung Medical Center patients who provided informed consent for the research. All participants in the UK Biobank provided their written informed consent.
Population characteristics	The basic characteristics of the patients are presented in Table 1. Data on risk factors including fundus photographs, sex, age, systolic blood pressure, diabetes, and hypertension were collected non-invasively from the EMRs. The remaining risk factors, that is, total cholesterol and HDL cholesterol, were measured from blood samples.
Recruitment	Data from the Samsung Medical Center was collected retrospectively and anonymized at the clinical data warehouse at the Samsung Medical Center. To test for external validity, we selected eligible, deidentified participants from approximately 500,000 participants in a prospective cohort, the UK Biobank. The patients of the Samsung Medical Center, who visited the Department of Ophthalmology, could have more underlying diseases than those in the UK Biobank, who were considered to be generally healthier participants. The racial differences can also affect the results.
Ethics oversight	Ethical approval for the study protocol was granted by the Institutional Review Board (No: 2016-05-561) at the Samsung Medical Center. The UK Biobank was approved by the National Research Ethnic Committee (REC reference 11/NW/0382).

Note that full information on the approval of the study protocol must also be provided in the manuscript.

Field-specific reporting

Please select the one below that is the best fit for your research. If you are not sure, read the appropriate sections before making your selection.

☒ Life sciences ☐ Behavioural & social sciences ☐ Ecological, evolutionary & environmental sciences

For a reference copy of the document with all sections, see nature.com/documents/nr-reporting-summary-flat.pdf

Life sciences study design

All studies must disclose on these points even when the disclosure is negative.

Sample size	Patients who visited the Samsung Medical Center between March 2010 and May 2016 with available fundus photographs were included in this study. We selected 2,543 patients with both fundus photographs and electronic medical records from 37,395 patients who had retinal fundus photographs from 2010 to 2016 in the Samsung Medical Center. To test for external validity, we selected eligible, deidentified participants from approximately 500,000 participants in a prospective cohort, the UK Biobank. The size of data may be sufficient to train the models retrospectively.
Data exclusions	Patients with pathology in the retina or vitreous, which affects the detection of microvascular changes in the fundus photographs (e.g., retinal detachment, macular degeneration, or vitreous hemorrhage), were excluded. The fundus images were reviewed by an ophthalmologist, and those with retinal diseases or of low quality were removed from the analysis. Additionally, diagnostic codes (International Classification of Diseases, 10th Revision, ICD-10, H33 and H353, H431) were applied to exclude fundus photographs of retinal diseases.
Replication	This study was externally validated with the UK Biobank.
Randomization	We used a total of 1,758 images for CVD cases and 1,760 images for non-CVD controls from the Samsung Medical Center for model development. For model validation, we used a total of 1,421 images for CVD cases and 1,533 images for non-CVD controls from the Samsung Medical Center, and 613 images for CVD cases and 10,685 images for controls from the UK Biobank. The non-CVD controls were randomly undersampled, corresponding to the number of cases in the development and internal validation sets, respectively.
Blinding	The investigators were blinded to group allocation during data collection.

Reporting for specific materials, systems and methods

We require information from authors about some types of materials, experimental systems and methods used in many studies. Here, indicate whether each material, system or method listed is relevant to your study. If you are not sure if a list item applies to your research, read the appropriate section before selecting a response.

Materials & experimental systems

n/a	Involved in the study
☒	☐ Antibodies
☒	☐ Eukaryotic cell lines
☒	☐ Palaeontology and archaeology
☒	☐ Animals and other organisms
☒	☐ Clinical data
☒	☐ Dual use research of concern

Methods

n/a	Involved in the study
☒	☐ ChIP-seq
☒	☐ Flow cytometry
☒	☐ MRI-based neuroimaging
